# TIMP2 ameliorates blood-brain barrier disruption in traumatic brain injury by inhibiting Src-dependent VE-cadherin internalization

**DOI:** 10.1172/JCI164199

**Published:** 2024-02-01

**Authors:** Jingshu Tang, Yuying Kang, Yujun Zhou, Nianying Shang, Xinnan Li, Hongyue Wang, Jiaqi Lan, Shuai Wang, Lei Wu, Ying Peng

**Affiliations:** State Key Laboratory of Bioactive Substances and Functions of Natural Medicines, Institute of Materia Medica, Chinese Academy of Medical Sciences and Peking Union Medical College, Beijing, China.

**Keywords:** Neuroscience, Vascular Biology, Molecular biology, Neurodegeneration

## Abstract

Blood-brain barrier (BBB) disruption is a serious pathological consequence of traumatic brain injury (TBI), for which there are limited therapeutic strategies. Tissue inhibitor of metalloproteinase-2 (TIMP2), a molecule with dual functions of inhibiting MMP activity and displaying cytokine-like activity through receptor binding, has been reported to inhibit VEGF-induced vascular hyperpermeability. Here, we investigate the ability of TIMP2 to ameliorate BBB disruption in TBI and the underlying molecular mechanisms. Both TIMP2 and AlaTIMP2, a TIMP2 mutant without MMP-inhibiting activity, attenuated neurological deficits and BBB leakage in TBI mice; they also inhibited junctional protein degradation and translocation to reduce paracellular permeability in human brain microvascular endothelial cells (ECs) exposed to hypoxic plus inflammatory insult. Mechanistic studies revealed that TIMP2 interacted with α_3_β_1_ integrin on ECs, inhibiting Src activation–dependent VE-cadherin phosphorylation, VE-cadherin/catenin complex destabilization, and subsequent VE-cadherin internalization. Notably, localization of VE-cadherin on the membrane was critical for TIMP2-mediated EC barrier integrity. Furthermore, TIMP2-mediated increased membrane localization of VE-cadherin enhanced the level of active Rac1, thereby inhibiting stress fiber formation. All together, our studies have identified an MMP-independent mechanism by which TIMP2 regulates EC barrier integrity after TBI. TIMP2 may be a therapeutic agent for TBI and other neurological disorders involving BBB breakdown.

## Abstract 2

<p>TIMP2 regulates an integrin &alpha;3/Src/VEC/Rac1 pathway to ameliorate blood-brain barrier disruption in traumatic brain injury.</p>

## Introduction

The blood-brain barrier (BBB) is a unique microvascular structure that separates neural tissue from the intravascular compartment to maintain brain homeostasis (1). Traumatic brain injury (TBI), the central nervous system (CNS) condition associated with severe BBB damage, is a major cause of disability and mortality among young adults (2). Following vascular damage induced by the initial mechanical impact, the secondary injury cascade further compromises BBB integrity, leading to brain edema and increased intracranial pressure, which result in poor clinical outcomes. BBB disruption is an early event in TBI with long-term effects (3). It causes serious neurological impairment (4), cognitive deficits (5), and other comorbidities, such as post-traumatic epilepsy (6). Thus, the BBB is an essential target for therapeutic intervention in TBI.

Brain capillary endothelial cells (ECs) are a vital component of the BBB. Specialized junctional complexes composed of tight junctions (TJs) and adherens junctions (AJs) are located between adjacent ECs and limit the entry of blood-borne factors into the brain parenchyma (7). During TBI pathology, including neuroinflammation (8) and cerebral hypoxia (9), the expression and localization of junctional complexes are altered, resulting in compromised vascular integrity (10). Vascular endothelial (VE)–cadherin, the key transmembrane component of endothelial AJs, is exclusively found in ECs (11). In addition to promoting homophilic adhesion between ECs, the cytoplasmic domain of VE-cadherin interacts with several anchoring proteins, such as α-/β-catenin, to form the VE-cadherin/catenin complex that transmits intracellular signals and regulates the dynamics of actin filaments (12–14). Exposure to inflammatory cytokines and oxidative stress components induces VE-cadherin tyrosine phosphorylation, which initiates VE-cadherin/catenin complex dissociation, uncoupling from the cytoskeleton, and subsequent VE-cadherin internalization (15, 16). The disassembly of AJs disrupts the overall junctional complex arrangement and increases vascular permeability (17).

MMPs are involved in the degradation of junctional complexes and basement membranes, resulting in the disruption of BBB integrity in the context of CNS diseases (18). Although tissue inhibitor of metalloproteinase-2 (TIMP2) is an endogenous inhibitor of MMP2 (19), experiments using AlaTIMP2, which lacks MMP-inhibiting activity (20), have shown that in addition to regulating the proteolytic activity of MMP2, TIMP2 displays diverse functions with cytokine activities. TIMP2 inhibits mitogen-driven capillary EC proliferation, migration, and angiogenesis by binding to specific cell surface receptors, such as ILGF-1R (21) or α_3_β_1_ integrin (22). The downstream signals that regulate antiangiogenic activity include SH2-containing protein tyrosine phosphatase-1–mediated (Shp-1–mediated) suppression of the receptor tyrosine kinases (RTKs) VEGFR2 (23) and FGFR1 (24), G_1_ growth arrest through induction of p27Kip1 (25), and enhanced cell surface expression of RECK via paxillin dephosphorylation (26). Notably, TIMP2 normalizes the VEGF-induced increase in vascular permeability by enhancing the association between VE-cadherin and the cytoskeleton via the Shp-1–cAMP/PKA pathway, indicating the potential application of TIMP2 in the treatment of diseases related to endothelial barrier breakdown (27). Additionally, MMP-independent mechanisms are also involved in TIMP2-mediated antitumorigenic activity (28, 29), neuronal differentiation (30), and neurite outgrowth (31). Given the broad biological functions regulated by TIMP2 through its nonproteolytic activity, it is essential to further characterize the underlying molecular mechanisms in specific physiological/pathophysiological conditions.

To date, TIMP2 has been linked to a variety of neuropathological conditions, displaying neuroprotective effects in dopaminergic cell death (32) and antiinflammatory effects in LPS-stimulated microglia (33). In animal models of CNS diseases, TIMP2 overexpression has been found to prevent the development of experimental autoimmune encephalomyelitis (34) and ischemic brain injury (35). In addition, systemic TIMP2 neutralization with anti-TIMP2 IgG alters spatial memory in young mice (36), while administration of recombinant TIMP2 reversed the cognitive deficits in aged mice (37). Although it has been recently reported that the regulatory effects of TIMP2 on cognition improvement are independent of its effects on MMPs, mechanistic exploration of its beneficial functions has mainly focused on neuronal activity and remains unclear (37). Considering the dual mechanisms of TIMP2 — i.e., inhibition of MMP2 activity and regulation of the biological function of ECs through cytokine activity — particularly in regulating VEGF stimulation–induced VE-cadherin translocation (27), TIMP2 may be superior to synthetic MMP inhibitors in maintaining BBB integrity. Therefore, in the present study, we explored whether TIMP2 plays a protective role in TBI by alleviating BBB disruption.

Here, we report an MMP-independent mechanism by which TIMP2 regulates EC barrier integrity in TBI. TIMP2 protects against BBB disruption and neurological deficits in TBI mice. In human brain microvascular ECs (HBMECs) exposed to hypoxia plus IL-1β, TIMP2 rescues the downregulation and mislocalization of junctional complexes. In terms of the mechanism, using AlaTIMP2 or EC-targeted α_3_ integrin knockdown in similar in vivo and in vitro TBI models, we demonstrate that TIMP2 binds to α_3_β_1_ integrin on ECs, mediating BBB integrity via nonproteolytic activity. TIMP2 subsequently inhibits Src activation to reduce VE-cadherin phosphorylation and VE-cadherin/catenin complex disassembly, contributing to VE-cadherin localization on the membrane. Notably, TIMP2-mediated stabilization of VE-cadherin at the membrane is required for Rac1 recruitment and activation, which contributes to actin cytoskeleton stabilization and overall junctional alignment. In conclusion, our results strongly support a role for TIMP2 in BBB protection and identify a TIMP2/α_3_ integrin/Src/VEC/Rac1 pathway that maintains the integrity of the EC barrier in TBI. We propose that TIMP2 is a potential therapeutic agent for rescuing BBB function in neurological disorders.

## Results

### TIMP2 improves neurological function and attenuates BBB disruption in mice after TBI.

**Figure 1:**
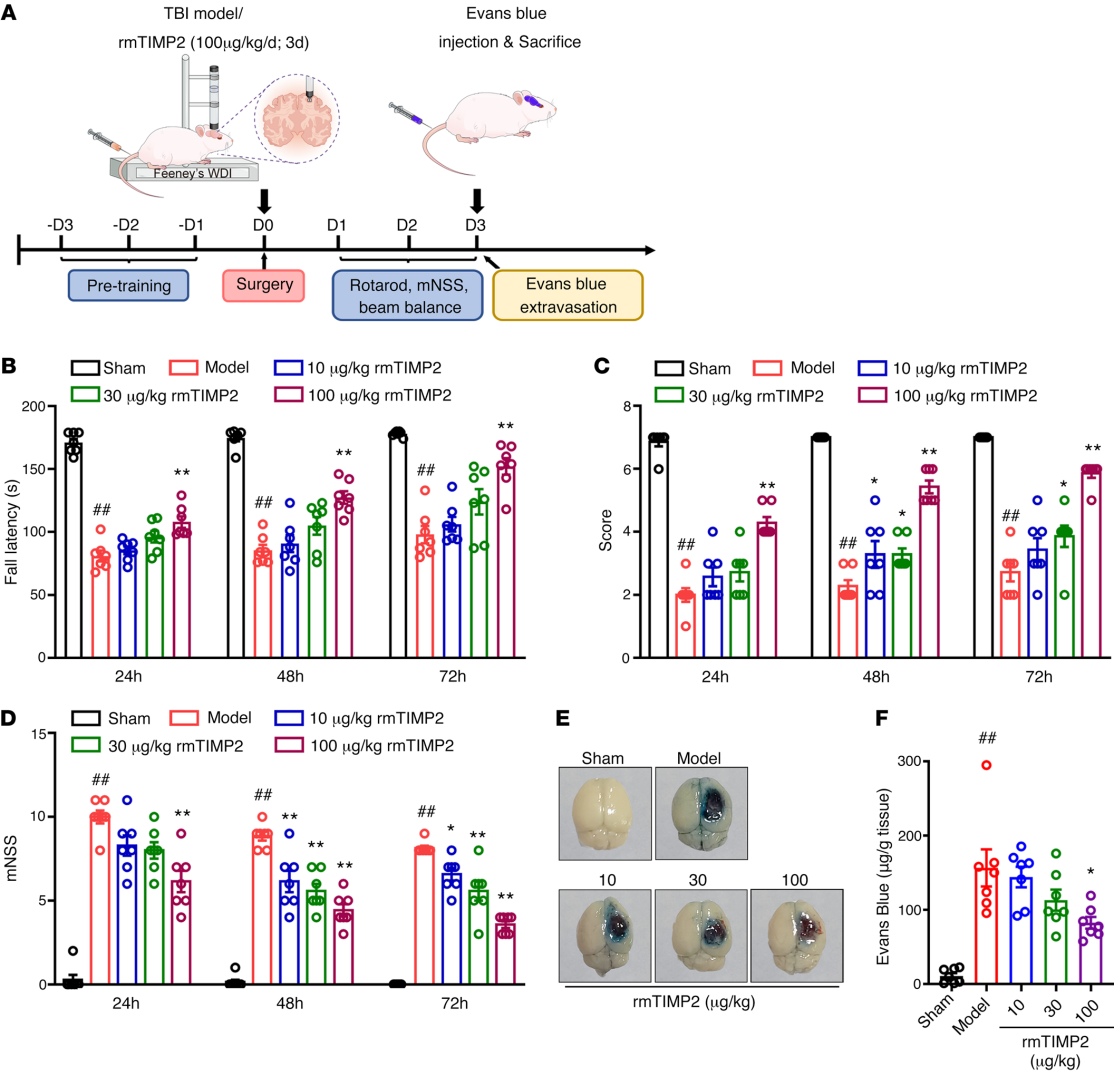
TIMP2 ameliorates neurological dysfunction and alleviates Evans blue extravasation in TBI. (**A**) Experimental scheme for TBI establishment, rmTIMP2 administration, neurological behavior assessment, and BBB integrity analysis in mice. WDI, weight drop injury. (**B**–**D**) TBI mice were intravenously given rmTIMP2 (10, 30, and 100 μg/kg) or PBS for 3 consecutive days, and neurological function was assessed at 24, 48, and 72 hours after TBI (*n* = 7 per group). (**B**) Fall latency of accelerated rotarod of mice from the indicated treatment groups. (**C**) Beam balance scores for mice from the indicated treatment groups. (**D**) mNSS of mice from the indicated treatment groups. (**E**) Representative images of brain tissues from the indicated treatment groups at 72 hours after TBI. The blue area indicates extravasation of Evans blue dye. (**F**) Quantification of leaked Evans blue dye in the ipsilateral cerebral hemisphere of mice from the indicated groups (*n* = 7 per group). ^##^*P* < 0.01 vs. sham group, *P < 0.05 and **P < 0.01 vs. model group, by 1-way ANOVA.

Mice subjected to experimental TBI exhibited neurological deficits. To investigate whether TIMP2 exerts protective effects against brain injury, TBI mice were intravenously given vehicle or different doses (10, 30, or 100 μg/kg) of purified recombinant murine TIMP2 (rmTIMP2) (Supplemental Figure 1A; supplemental material available online with this article; https://doi.org/10.1172/JCI164199DS1) for 3 consecutive days, and neurological function was assessed (Figure 1A). Significant impairments in motor function in the rotarod test and beam walk test were observed in the vehicle-treated TBI group, as manifested by reductions in exercise time and hind-limb motor scores, respectively (Figure 1, B and C). Treatment with 10 μg/kg and 30 μg/kg rmTIMP2 did not improve the performance of TBI mice in the rotarod test; 100 μg/kg rmTIMP2 significantly reversed the reduction in fall latency at the indicated times after TBI (Figure 1B). In addition, hind-limb scores were dose-dependently increased in the rmTIMP2-treated groups compared with the vehicle-treated groups at the indicated times (Figure 1C). The above results suggest that TIMP2 can rescue TBI-induced motor impairment.

We further used the modified neurological severity score (mNSS) scale, the most commonly used neurological scale in animal studies of neurodegenerative diseases, to comprehensively assess the effect of rmTIMP2 on neurological function in TBI mice. As shown in Figure 1D, the mNSS was significantly lower in the 30 μg/kg and 100 μg/kg rmTIMP2-treated TBI mice than in the vehicle-treated TBI mice at 48 hours and 72 hours after TBI, demonstrating that post-trauma administration of TIMP2 improves the recovery of neurological function.

Next, we evaluated the effect of rmTIMP2 on the BBB. Evans blue extravasation was examined 5 hours and 3 days after injury, and the time points were determined based on previous findings that there is a biphasic increase in BBB permeability following TBI, peaking at 4–6 hours and 2–3 days, respectively (38). As shown graphically in Figure 1E and Supplemental Figure 2A, and quantitatively in Figure 1F and Supplemental Figure 2B, Evans blue extravasation in the ipsilateral hemispheric parenchyma of TBI mice was significantly increased at both phases. Although rmTIMP2 treatment failed to inhibit the early opening of BBB (Supplemental Figure 2), the profound increase in Evans blue leakage in the second opening phase was markedly attenuated by 100 μg/kg rmTIMP2 treatment (Figure 1, E and F), indicating that the administration of TIMP2 ameliorates TBI-induced BBB hyperpermeability.

### TIMP2 maintains endothelial barrier integrity under hypoxic plus inflammatory insult.

**Figure 2:**
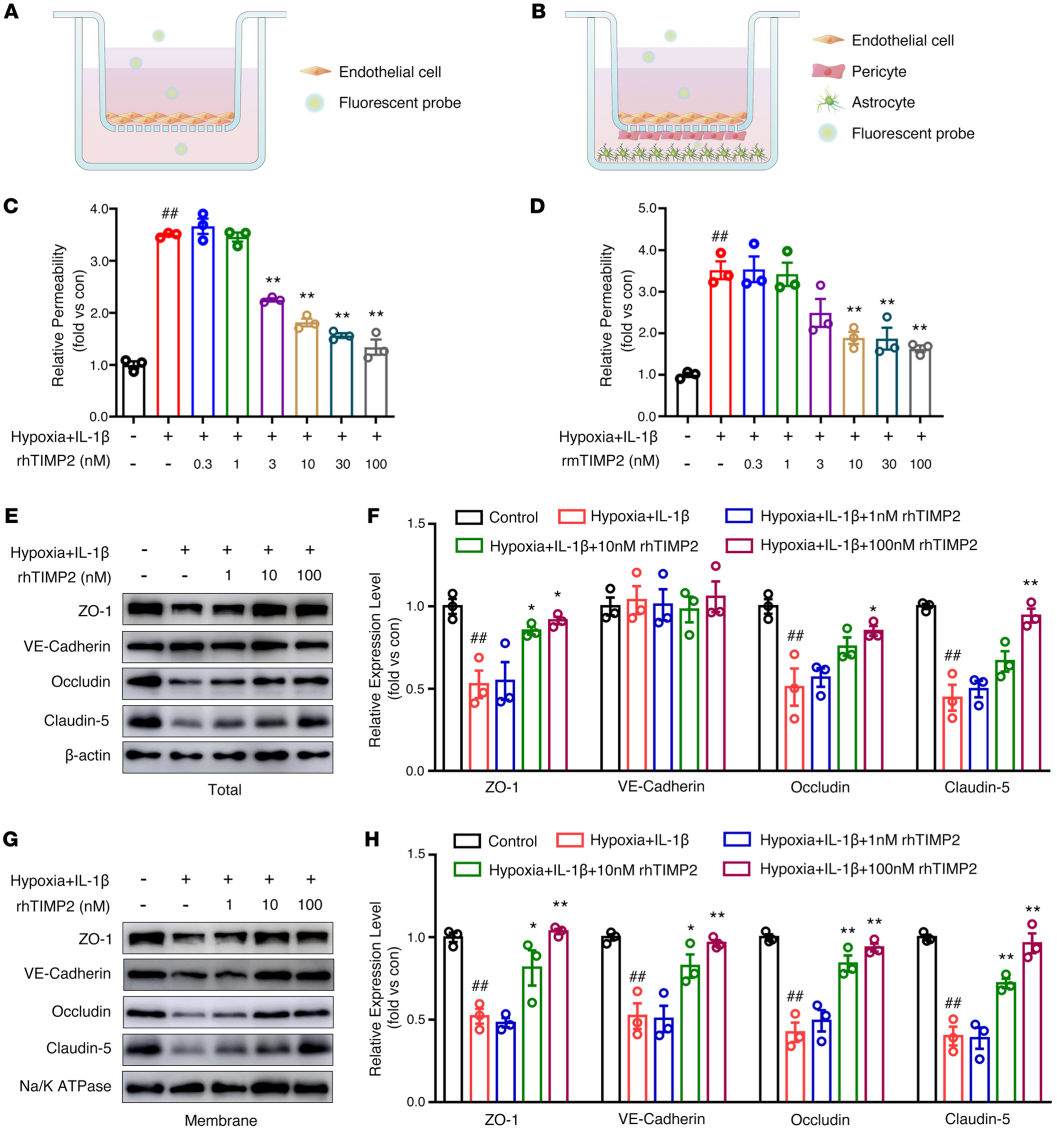
TIMP2 attenuates EC barrier leakage and JP disruption induced by hypoxia plus IL-1β insult. (**A**–**D**) Cells were treated with rhTIMP2, rmTIMP2, or PBS, and then subjected to hypoxia plus IL-1β insult for 24 hours. Paracellular permeability was determined by measurement of the concentration of 70 kDa FITC-dextran leaking from the luminal to abluminal. (**A**) Illustration of the in vitro BBB model composed of an HBMEC monolayer seeded on top of a cell culture insert. (**B**) Illustration of the in vitro 3D BBB model composed of primary cells, including BMECs seeded on the apical side, pericytes seeded on the underside of the insert, and astrocytes seeded on the plate bottom. (**C**) The effects of the rhTIMP2 dose range on the paracellular permeability of HBMECs. (**D**) The effects of the rmTIMP2 dose range on the paracellular permeability of primary BMECs. (**E**–**H**) HBMECs treated with rhTIMP2 or PBS were subjected to hypoxia plus IL-1β insult for 24 hours. Western blot analysis (**E**) and quantification (**F**) of the indicated proteins in whole-cell extracts. Western blot analysis (**G**) and quantification (**H**) of the indicated proteins in the membrane fraction. Data represent 3 independent experiments. ^##^*P* < 0.01 vs. control group, *P < 0.05 and **P < 0.01 vs. hypoxia plus IL-1β group, by 1-way ANOVA.

To investigate the role of TIMP2 in BBB protection, we first established an in vitro BBB model composed of a monolayer of HBMECs (Figure 2A) or triple culture of primary mouse microvessel ECs, pericytes, and astrocytes (Figure 2B). The purity of the primary cells was determined by specific markers, including vWF for the ECs, PDGFRβ for pericytes, and GFAP for astrocytes (Supplemental Figure 3). Since hypoxia and inflammation are the key factors driving secondary injury after TBI and IL-1β is the proinflammatory cytokine most strongly involved in TBI (9, 39), we exposed the in vitro BBB model to hypoxia plus 20 ng/mL IL-1β for 24 hours to mimic in vivo pathological conditions. Using FITC-dextran leakage to evaluate transendothelial permeability, we applied various concentrations of recombinant human TIMP2 (rhTIMP2) (Supplemental Figure 4A) and rmTIMP2 to the BBB model exposed to hypoxia plus IL-1β injury. Compared with control treatment, hypoxic plus inflammatory insult significantly increased the diffusion of fluorescent tracers across the luminal-to-abluminal barrier, while exogenous addition of rhTIMP2 (0.3–100 nM) dose-dependently reversed the enhanced paracellular permeability of the EC barrier (Figure 2C). Similar effects were also observed in the triple-coculture BBB model treated with rmTIMP2 (0.3–100 nM) (Figure 2D). These results suggest that TIMP2 plays an important role in regulating endothelial barrier integrity.

We next examined the effects of TIMP2 on the expression and distribution of junctional proteins (JPs), which are essential for maintaining endothelial tightness. Western blot analysis revealed that 100 nM rhTIMP2 markedly abrogated the downregulation of TJ protein (ZO-1, occludin, and claudin-5) expression following exposure to hypoxia plus IL-1β (Figure 2, E and F). Notably, although the total expression level of the AJ protein VE-cadherin remained unchanged, by purifying the plasma membrane with a biotin-avidin system, we observed that VE-cadherin shifted from the membrane to the cytosolic fraction. However, upon the addition of rhTIMP2, VE-cadherin translocation in HBMECs was greatly reduced, indicating that rhTIMP2 reversed the reduction in functional VE-cadherin levels after hypoxic plus inflammatory injury (Figure 2, G and H).

### TIMP2 regulates the endothelial barrier through an MMP-independent mechanism.

**Figure 3:**
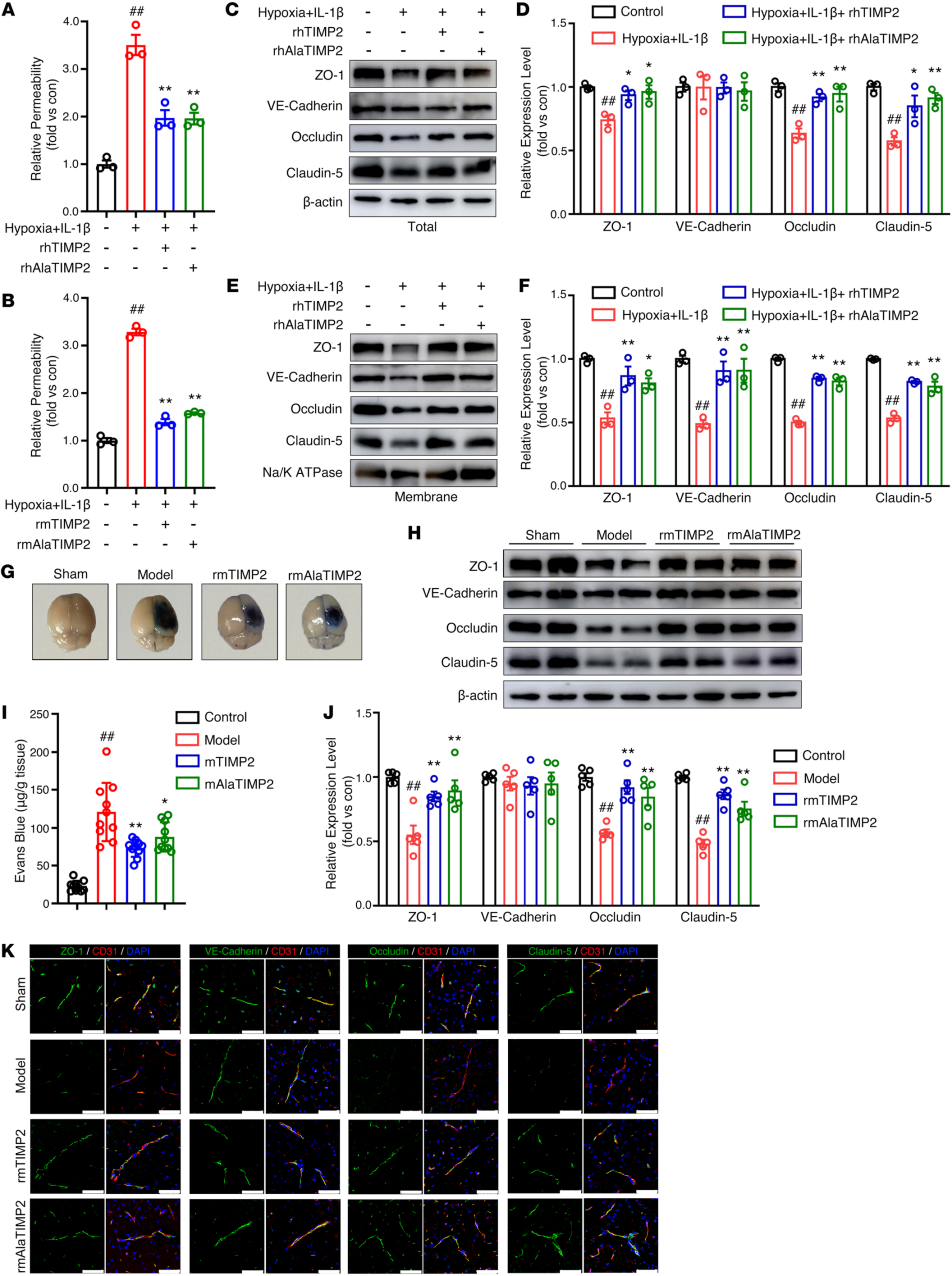
TIMP2 regulates the EC barrier and JP expression in TBI in an MMP-independent manner. (**A**–**F**) Cultured cells treated with rhTIMP2, rhAlaTIMP2, rmTIMP2, rmAlaTIMP2, or PBS were subjected to hypoxia plus IL-1β injury for 24 hours. Transwell permeability assays were performed to assess EC barrier integrity in an in vitro BBB model consisting of an HBMEC monolayer (**A**) and an in vitro 3D BBB model consisting of primary BMECs, pericytes, and astrocytes (**B**). (**C** and **D**) Western blot analysis (**C**) and quantification (**D**) of the indicated proteins in whole-cell extracts. (**E** and **F**) Western blot analysis (**E**) and quantification (**F**) of the indicated proteins in membrane fractions. Data represent 3 independent experiments. ^##^*P* < 0.01 vs. control group, *P < 0.05 and **P < 0.01 vs. hypoxia plus IL-1β group, by 1-way ANOVA. (**G**–**K**) TBI mice were intravenously given 100 μg/kg rmTIMP2, 100 μg/kg rmAlaTIMP2, or PBS for 3 consecutive days. Representative images (**G**) and quantification (**H**) of Evans blue dye leaking into the ipsilateral cerebral hemisphere at 72 hours after TBI (*n* = 10 per group). Microvessels were isolated and subjected to Western blot analysis (**I**) and quantification (**J**) of the indicated proteins (*n* = 5 per group). (**K**) Immunofluorescence staining analysis of the indicated proteins in the ipsilateral hemispheric brain (*n* = 5 per group). Scale bars: 50 μm. ^##^*P* < 0.01 vs. sham group, *P < 0.05 and **P < 0.01 vs. model group, by 1-way ANOVA.

Previous studies have revealed that in addition to inhibiting MMPs, TIMP2 displays potent angio-inhibitory and antitumorigenic activity in vivo through nonproteolytic mechanisms (28). To determine whether the MMP-independent mechanism is involved in TIMP2-regulated EC barrier integrity, we generated AlaTIMP2, a mutant of TIMP2 lacking MMP-inhibiting activity, which provides steric hindrance to prevent inhibition of MMPs by TIMP2 (20). The purity of rhAlaTIMP2 and rmAlaTIMP2 was identified by SDS-PAGE (Supplemental Figure 1B and Supplemental Figure 4B). In the monolayer BBB model exposed to hypoxic plus inflammatory insult, 100 nM rhTIMP2 and 100 nM rhAlaTIMP2 attenuated FITC-dextran leakage to the same extent (Figure 3A). Similar results were also observed in the triple-coculture BBB model with exogenous addition of rmTIMP2 and rmAlaTIMP2 (Figure 3B). Moreover, there was no significant difference between rhTIMP2 and rhAlaTIMP2 in their ability to ameliorate the hypoxic plus inflammatory insult–induced reduction in the total expression of JPs (Figure 3, C and D) or the decrease in JP localization on the membrane (Figure 3, E and F).

We further investigated the importance of TIMP2-mediated MMP-independent activity in maintaining BBB integrity in TBI. Postinjury administration of 100 μg/kg rmTIMP2 and its mutant form rmAlaTIMP2 protected TBI mice from neurological deficits, improving motor performance in the rotarod test (Supplemental Figure 5A) and the beam balance test (Supplemental Figure 5B) and decreasing the mNSS (Supplemental Figure 5C). Importantly, compared with vehicle treatment, 100 μg/kg rmAlaTIMP2 significantly reduced Evans blue extravasation in the injured brain (Figure 3, G and H). Western blotting was performed to analyze the expression of JPs in microvessels isolated from tissue in the ipsilateral hemisphere. We observed that rmTIMP2, with or without MMP-inhibitory activity, markedly restored the expression levels of JPs compared with the vehicle-treated group (Figure 3, I and J). Consistent with the Western blot results, immunostaining analysis revealed that rmTIMP2 and rmAlaTIMP2 rescued the loss of JPs located in proximity to the brain injury site (Figure 3K). Together, these data from in vitro and in vivo models indicate that TIMP2 preserves BBB integrity after TBI via an MMP-independent mechanism.

### TIMP2 interacts with α_3_β_1_ integrin to regulate the endothelial barrier.

**Figure 4:**
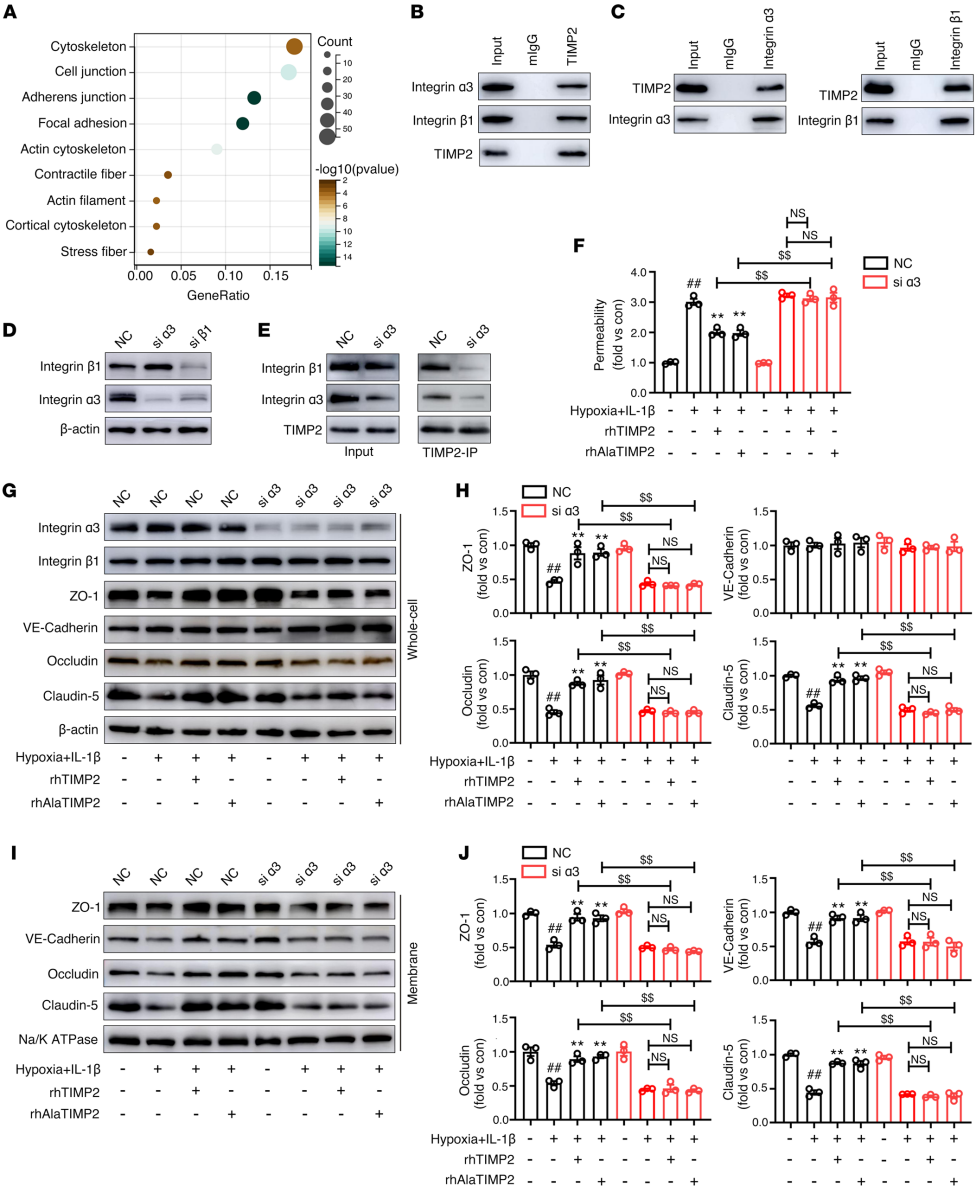
TIMP2 interacts with α_3_β_1_ integrin to regulate the EC barrier in vitro. (**A**) The Gene Ontology terms of the cell component category enrichment of proteins interacting with TIMP2 in HBMECs. (**B**) Total cell lysates from HBMECs were extracted and subjected to TIMP2 IP, followed by Western blot analysis for the indicated proteins. (**C**) Total cell lysates from HBMECs were extracted and subjected to β_1_ integrin or α_3_ integrin IP assays, followed by Western blot analysis for the indicated proteins. (**D**) HBMECs were treated with siRNA against β_1_ integrin or α_3_ integrin, followed by Western blot analysis for the indicated proteins. (**E**) HBMECs treated with siRNA against α_3_ integrin were used for TIMP2 IP assays, followed by Western blot analysis for the indicated proteins. (**F**–**J**) HBMECs transfected with siRNA against α_3_ integrin were treated with rhTIMP2, rhAlaTIMP2, or PBS, and then subjected to hypoxia plus IL-1β injury for 24 hours. Transwell permeability assays were performed to assess EC barrier integrity in an in vitro BBB model (**F**). Western blot analysis (**G**) and quantification (**H**) of the indicated proteins in whole-cell extracts. Western blot analysis (**I**) and quantification (**J**) of the indicated proteins in membrane fractions. Data represent 3 independent experiments. ^##^*P* < 0.01 vs. control group, **P < 0.01 vs. hypoxia plus IL-1β group, ^$$^*P* < 0.01, by 1-way ANOVA.

To understand the mechanism underlying the function of TIMP2 in maintaining EC barrier integrity, we investigated the protein complexes that interact with TIMP2. Total HBMEC lysates were subjected to immunoprecipitation (IP) using an anti-TIMP2 antibody or control mIgG, and then liquid chromatography–tandem mass spectrometry (LC-MS/MS) was performed to identify the coprecipitated proteins. Proteins immunoprecipitated with the anti-TIMP2 antibody were compared with those immunoprecipitated with mIgG. Only the proteins identified in the TIMP2 group were considered specific interacting partners (Supplemental Table 1), which were functionally annotated using Gene Ontology Cellular Component analysis. The results revealed that TIMP2 interacts with multiple plasma membrane proteins, such as cell junction, AJ, and focal adhesion proteins, and cytoskeletal components, including the actin cytoskeleton, cortical cytoskeleton, and stress fibers (Figure 4A).

TIMP2 is a secreted protein that exerts cytokine activity by interacting with membrane receptors. Therefore, we attempted to identify the specific receptor for TIMP2 in ECs. Our IP-MS results revealed that focal adhesion is a major component of the TIMP2 interactome in HBMECs (Figure 4A). Cell surface integrins are membrane components of the focal adhesion complex that not only mediate outside-in signaling but also play significant roles in regulating AJ stabilization and cytoskeleton organization. Thus, we chose to focus on α_3_β_1_ integrin in subsequent studies, as it is the only integrin family member identified by TIMP2 IP-MS. Western blot analysis revealed that TIMP2 could pull down both α_3_ integrin and β_1_ integrin (Figure 4B). In parallel, both α_3_ integrin and β_1_ integrin IP pulled down TIMP2, confirming that TIMP2 forms a ternary complex with α_3_β_1_ integrin on HBMECs (Figure 4C).

We next sought to determine whether α_3_β_1_ integrin is required for TIMP2-mediated EC barrier integrity. The protein expression of α_3_ integrin and β_1_ integrin was significantly downregulated in HBMECs transfected with α_3_ integrin– and β_1_ integrin–specific siRNAs, respectively. Notably, silencing β_1_ integrin decreased the expression of α_3_ integrin, while alterations in the expression level of α_3_ integrin had little effect on β_1_ integrin (Figure 4D). Furthermore, knockdown of α_3_ integrin attenuated the interaction between TIMP2 and β_1_ integrin (Figure 4E), suggesting that the binding of TIMP2 to the α_3_ subunit is essential for the interaction between TIMP2 and α_3_β_1_ integrin. Since β_1_ integrin can form heterodimers with all α subunits, to eliminate the potential effects of β_1_ integrin on the expression levels of other α subunits, we chose to knock down α_3_ integrin for subsequent studies on the mechanism of TIMP2.

**Figure 5:**
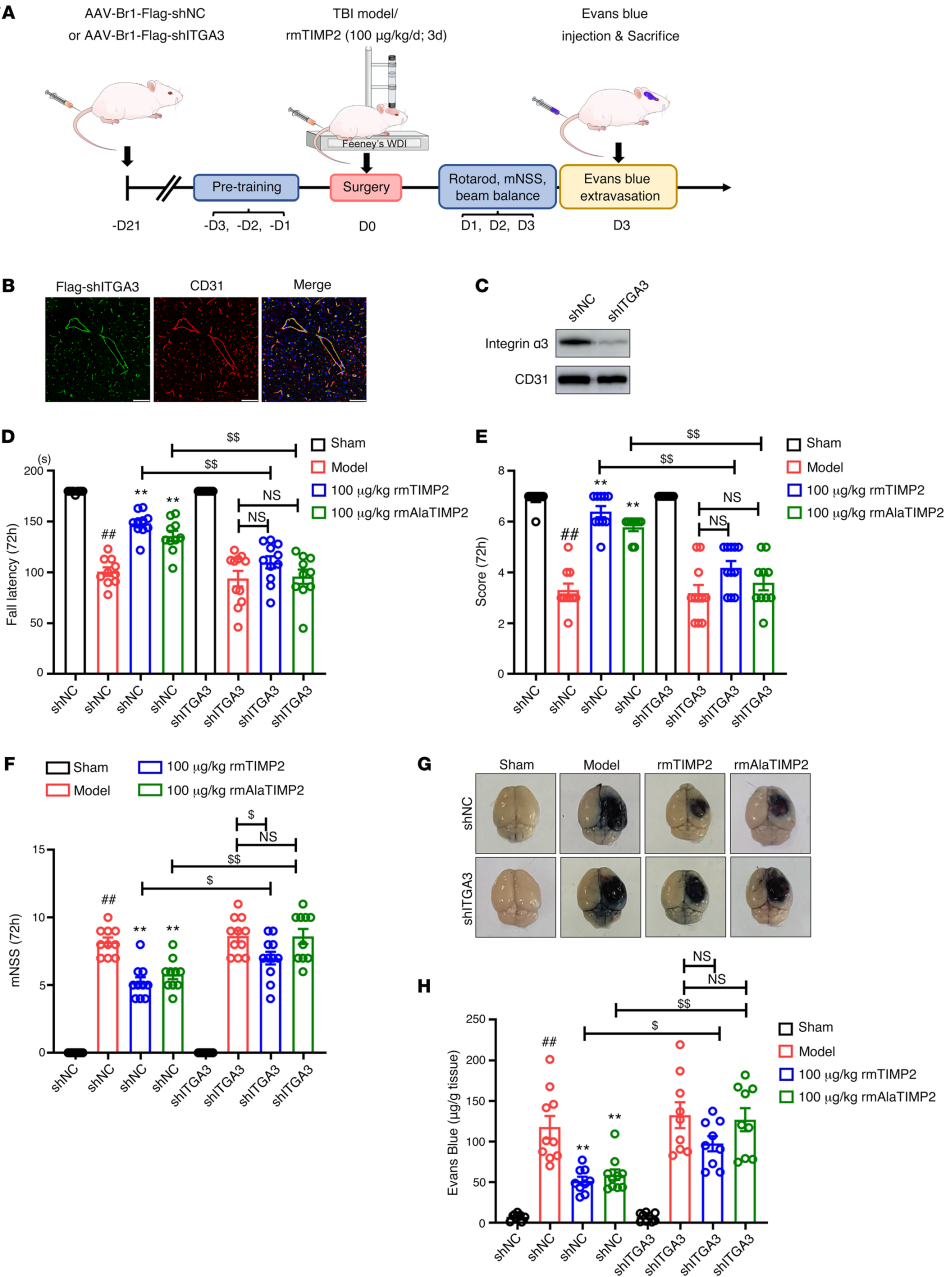
TIMP2 interacts with α_3_β_1_ integrin to alleviate TBI-induced BBB disruption. (**A**) Experimental scheme for AAV-BR1 targeting cerebrovascular knockdown of α_3_ integrin, establishment of TBI, and analysis of BBB integrity in mice. (**B**–**H**) Mice were intravenously injected with AAV-BR1-FLAG-shITGA3 or AAV-BR1-FLAG-shNC. Three weeks after AAV injection, experimental TBI was established in mice, which were treated with 100 μg/kg rmTIMP2, 100 μg/kg rmAlaTIMP2, or PBS, and neurological function was assessed at 72 hours after TBI. (**B**) FLAG-shITGA3–transduced cells were analyzed by immunofluorescence staining using FLAG (green) and the endothelial marker CD31 (red) in brain cortex 21 days after AAV injection. Scale bars: 75 μm. (**C**) Microvasculature isolated from brain 21 days after AAV injection and subjected to Western blot analysis for the indicated proteins. Neurological function, including fall latency of accelerated rotarod (**D**), beam balance (**E**), and mNSS (**F**), was assessed at 72 hours after TBI (*n* = 9–11 per group). (**G** and **H**) Representative images (**G**) and quantification (**H**) of Evans blue dye leakage into the ipsilateral cerebral hemisphere at 72 hours after TBI (*n* = 9–10 per group). ^##^*P* < 0.01 vs. sham group, **P < 0.01 vs. model group, ^$^*P* < 0.05, ^$$^*P* < 0.01, by 1-way ANOVA.

Using an in vitro BBB model to assess paracellular permeability, we observed that neither rhTIMP2 nor rhAlaTIMP2 could inhibit hypoxic plus inflammatory injury–induced FITC-dextran leakage in α_3_ integrin–knockdown cells (Figure 4F). In addition, knockdown of α_3_ integrin was sufficient to abolish the rescuing effect of TIMP2 on the reduction in JP expression (Figure 4, G and H) and the mislocalization of JPs (Figure 4, I and J). To confirm that the binding to α_3_ integrin on brain ECs is critical for the ability of TIMP2 to reduce BBB damage in TBI, a brain microvasculature EC-targeted adeno-associated virus (AAV2 displaying the NRGTEWD peptide), termed AAV-BR1, was used to specifically knock down α_3_ integrin in brain ECs (40) (Figure 5A). Immunofluorescence staining showed that 3 weeks after intravenous injection of AAV-BR1-FLAG-shITGA3 or AAV-BR1-FLAG-shNC (negative control) into mice, the expression of AAV-BR1-FLAG was restricted to the brain vasculature, as reflected by its precise colocalization with CD31 (Figure 5B), indicating that AAV-BR1 was successfully transduced into brain ECs. In addition, a significant reduction in the expression of α_3_ integrin was observed in microvessels purified from mouse brains transduced with AAV-BR1-FLAG-shITGA3, confirming the efficiency of α_3_ integrin knockdown (Figure 5C). Based on the results of AAV-mediated EC-specific α_3_ integrin knockdown in vivo, we induced experimental TBI in mice 3 weeks after AAV injection and then performed behavioral assessments and analyzed BBB integrity. As expected, in comparison with AAV-shNC–injected mice, EC-specific α_3_ knockdown in mice significantly reversed the effects of rmTIMP2 and rmAlaTIMP2 on attenuation of neurological deficits, as reflected by poorer performance on the rotarod and beam balance tests and a decreased mNSS (Figure 5, D–F, and Supplemental Figure 6). In addition, the beneficial effects of rmTIMP2 and rmAlaTIMP2 on BBB integrity were also blocked by EC-specific α_3_ knockdown, since the Evans blue extravasation could not be attenuated in AAV-shITGA3–injected TBI mice as it was in TBI mice injected with AAV-shNC (Figure 5, G and H). It is worth noting that in α_3_ integrin siRNA–treated HBMECs and EC-specific α_3_-knockdown mice, there was no significant difference between the rTIMP2/rAlaTIMP2 group and the corresponding model group. Collectively, these results strongly demonstrate that binding to α_3_β_1_ integrin is specifically required for TIMP2-mediated EC barrier integrity in TBI.

### TIMP2 inhibits Src activation to modulate VE-cadherin phosphorylation.

**Figure 6:**
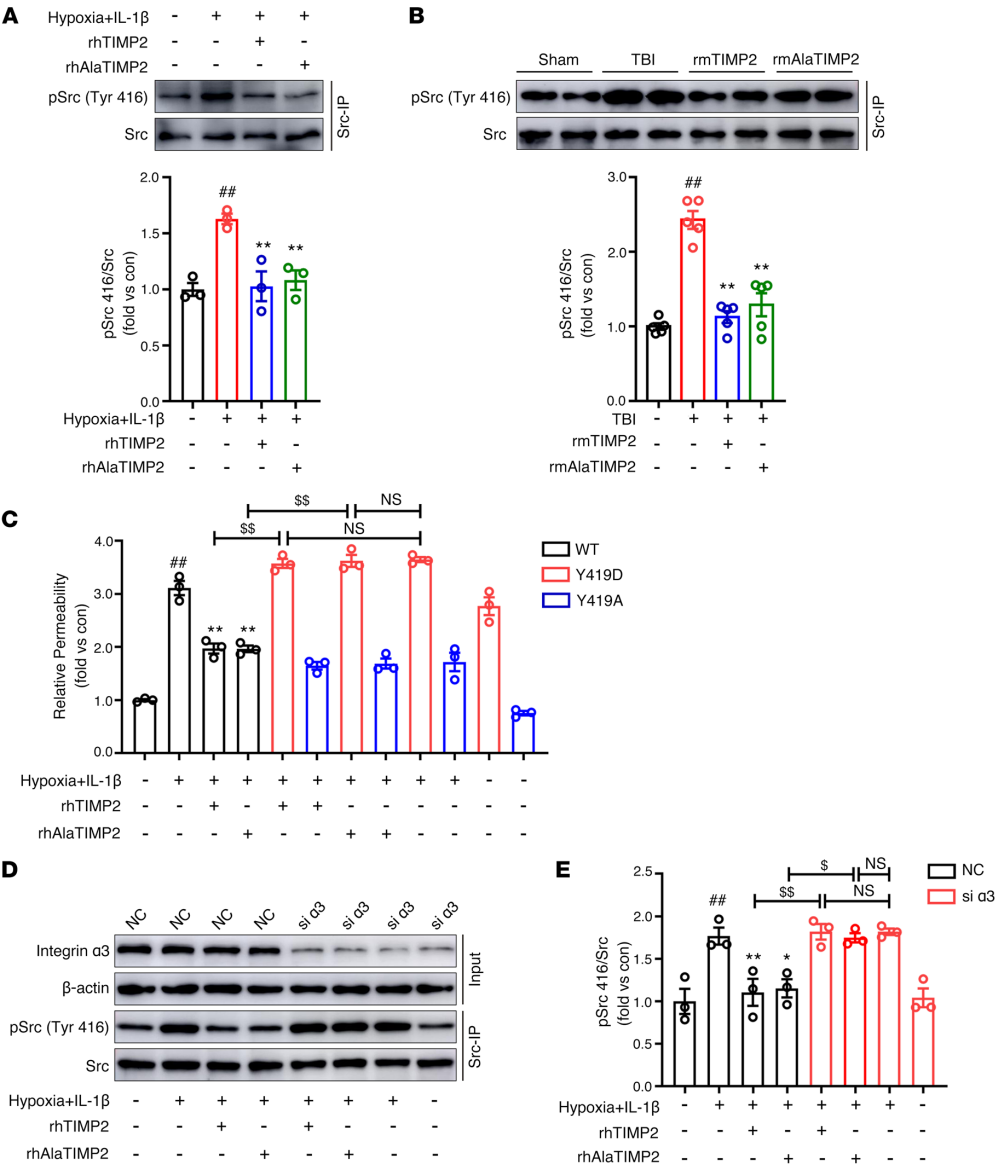
TIMP2 inhibits TBI-induced Src activation. (**A**) HBMECs treated with rhTIMP2, rhAlaTIMP2, or PBS were subjected to hypoxia plus IL-1β injury for 24 hours. Total cell lysates were subjected to Western blot analysis for p-Src (Tyr416) and Src. Data represent 3 independent experiments. ^##^*P* < 0.01 vs. control group, **P < 0.01 vs. hypoxia plus IL-1β group, by 1-way ANOVA. (**B**) TBI mice were treated with rmTIMP2, rmAlaTIMP2, or PBS for 3 days. Western blot analysis and quantification of p-Src (Tyr416) and Src in the ipsilateral cerebral hemisphere (*n* = 5 per group). ^##^*P* < 0.01 vs. sham group, **P < 0.01 vs. model group, by 1-way ANOVA. (**C**) HBMECs transfected with WT, Y419D, or Y419A Src were treated with rhTIMP2, rhAlaTIMP2, or PBS, and then subjected to hypoxia plus IL-1β injury for 24 hours. Transwell permeability assays were performed to assess EC barrier integrity. (**D** and **E**) HBMECs transfected with siRNA against α_3_ integrin were treated with rhTIMP2, rhAlaTIMP2, or PBS, and then subjected to hypoxia plus IL-1β injury for 24 hours. Western blot analysis (**D**) and quantification (**E**) of p-Src (Tyr416) and Src. Data represent 3 independent experiments. ^##^*P* < 0.01 vs. control group, *P < 0.05 and **P < 0.01 vs. hypoxia plus IL-1β group, ^$^*P* < 0.05, ^$$^*P* < 0.01, by 1-way ANOVA.

Previous studies have revealed that proinflammatory stimuli induce Src activation, leading to EC barrier hyperpermeability (41). Src kinase serves as a downstream effector of integrin family members (42); therefore, we sought to investigate whether the regulation of Src activation is involved in the effects of TIMP2 on EC barrier integrity in TBI. Exogenous addition of rhTIMP2 and rhAlaTIMP2 attenuated the phosphorylation of Src at Tyr416 in HBMECs under hypoxic plus inflammatory injury (Figure 6A). Consistently, we also observed that Src activation was inhibited in TBI mice following rmTIMP2 and rmAlaTIMP2 treatment (Figure 6B). In addition, as previously reported (43), rTIMP2 increased the phosphorylation level of Src at Tyr527, the negative regulatory site of Src kinase activity (Supplemental Figure 7, A and B). To further confirm that inhibition of Src phosphorylation underlies the barrier-protecting function of TIMP2 in ECs, we performed site-directed mutagenesis to generate a kinase-dead Src variant (Y419A) (44) and a constitutively active Src variant (Y419D) (45). Evidently, compared with HBMECs overexpressing wild-type Src (WT), the introduction of the Y419D construct completely eliminated the inhibitory effect of TIMP2 on EC paracellular permeability, as reflected by a robust increase in FITC-dextran leakage. In contrast, HBMECs expressing the phosphorylation-resistant variant Y419A showed little change in TIMP2-mediated paracellular permeability (Figure 6C). Furthermore, silencing α_3_ integrin fully abolished the inhibitory effects of TIMP2 and its mutant on Src activation in HBMECs (Figure 6, D and E, and Supplemental Figure 7C). Together, these results suggest that Src activation is downstream of α_3_ integrin, playing a crucial role in the regulatory effects of TIMP2 on EC barrier integrity.

**Figure 7:**
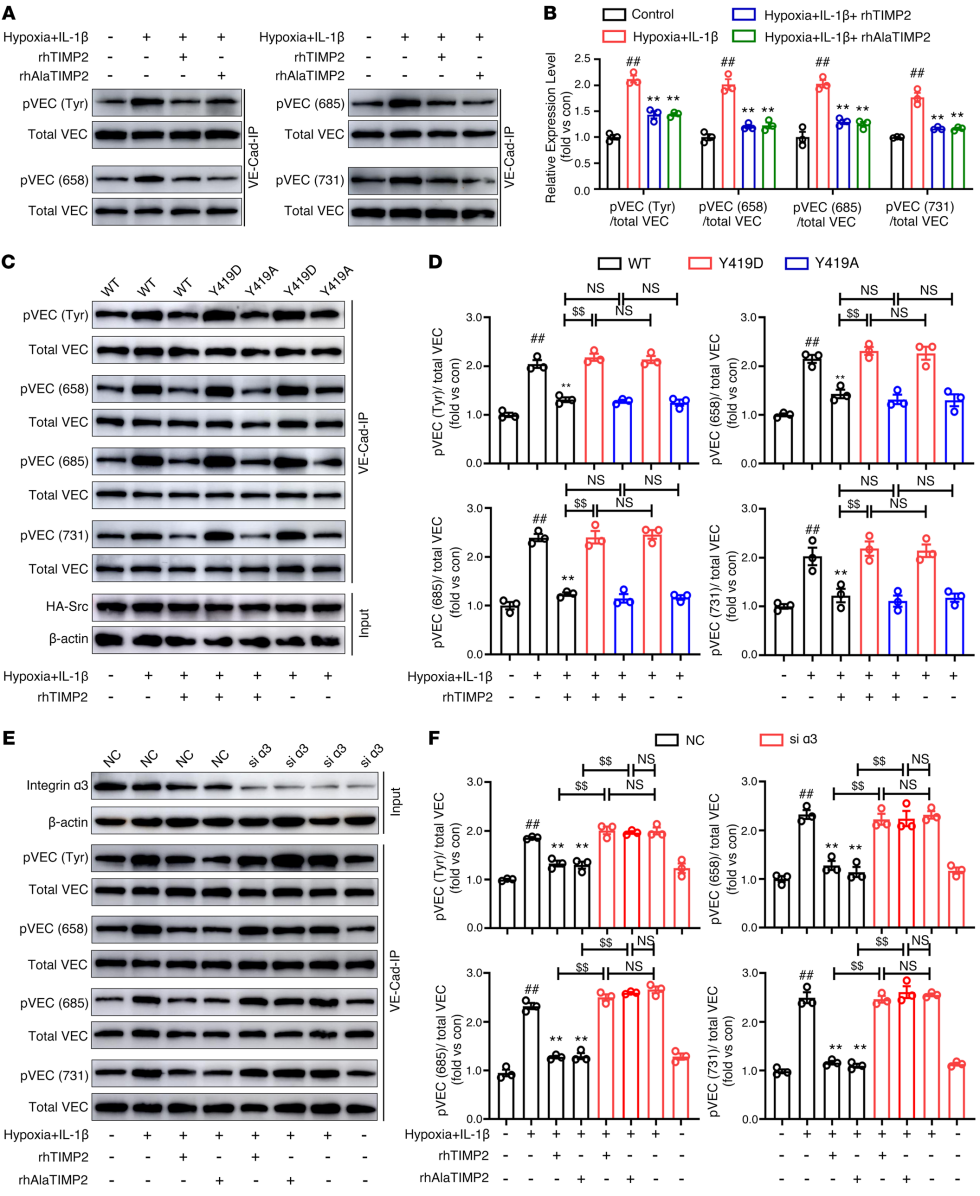
TIMP2 decreases hypoxic plus IL-1β injury–induced VE-cadherin phosphorylation through Src inhibition. (**A** and **B**) HBMECs were treated with rhTIMP2, rhAlaTIMP2, or PBS, and then subjected to hypoxia plus IL-1β injury for 24 hours. Western blot analysis (**A**) and quantification (**B**) of p–VE-cadherin at the indicated sites and total VE-cadherin. (**C** and **D**) HBMECs transfected with WT, Y419D, or Y419A Src were treated with rhTIMP2 or PBS, and then subjected to hypoxia plus IL-1β injury for 24 hours. Western blot analysis (**C**) and quantification (**D**) of p–VE-cadherin at the indicated sites and total VE-cadherin. (**E** and **F**) HBMECs transfected with siRNA against α_3_ integrin were treated with rhTIMP2, rhAlaTIMP2, or PBS, and then subjected to hypoxia plus IL-1β injury for 24 hours. Western blot analysis (**E**) and quantification (**F**) of p–VE-cadherin at the indicated sites and total VE-cadherin. Data represent 3 independent experiments. ^##^*P* < 0.01 vs. control group, **P < 0.01 vs. hypoxia plus IL-1β group, ^$$^*P* < 0.01, by 1-way ANOVA.

VE-cadherin is a critical structural component for brain vasculature integrity, playing an essential role in junctional complex stabilization and actin cytoskeletal remodeling (46). Src activation induces tyrosine phosphorylation in the cytosolic tail of VE-cadherin, leading to barrier function disruption. Constitutively active Src phosphorylated VE-cadherin on tyrosines 658 and 731, which are critical for developing a restrictive monolayer (16, 47, 48). Phosphorylation of VE-cadherin at Y685 by Src activation is required for eNOS-induced vascular barrier disruption in retinopathy (49) and angiotensin II–induced pulmonary microvascular endothelial barrier injury (50). To gain further insight into the downstream mechanism of TIMP2-mediated Src inhibition, we first investigated whether VE-cadherin phosphorylation is critical for the ability of TIMP2 to maintain the EC barrier in the hypoxia plus IL-1β model. Performing VE-cadherin IP followed by assessment of total tyrosine phosphorylation using a phosphotyrosine antibody, we found that hypoxia plus inflammatory injury resulted in increased total tyrosine phosphorylation of VE-cadherin in HBMECs, which was markedly inhibited by rhTIMP2 and rhAlaTIMP2 treatments (Figure 7, A and B). Next, we further performed VE-cadherin IP and identified that TIMP2 regulated phosphorylation of VE-cadherin at Y658, Y685, and Y731 (Figure 7, A and B). To confirm the critical sites involved in TIMP2-mediated barrier function, we introduced different combinations of VE-cadherin phosphorylation mimic variants, including Y685E, Y658&731E, and Y658&Y685&731E (VEC-3mu). Interestingly, compared with the corresponding model group, neither Y685E nor Y658&731E could completely reverse the TIMP2 inhibitory effects on paracellular permeability (Supplemental Figure 8, A and B), whereas HBMECs expressing VE-cadherin with combinatorial Y658&Y685&731E mutation sites failed to respond to TIMP2-mediated inhibition effects on hyperpermeability (Supplemental Figure 8C). In addition, the expression of VEC-3mu strongly inhibited the regulatory effect of TIMP2 on TJ expression in both the cell lysate and the membrane fraction (Supplemental Figure 8, D–G). Collectively, these results indicate a critical role of VE-cadherin phosphorylation in TIMP2-mediated endothelial barrier integrity.

Next, to investigate whether the inhibition of Src phosphorylation downstream of TIMP2 signaling mediated VE-cadherin phosphorylation, we used overexpression of WT, Y419D, or Y419A Src constructs in HBMECs. Western blotting revealed that, compared with overexpression of WT Src, overexpression of the Y419D mutant, but not the Y419A mutant, greatly reversed the inhibitory effect of rhTIMP2 and rhAlaTIMP2 on VE-cadherin phosphorylation. Furthermore, in HBMECs expressing Src Y419D, there was no significant difference between the TIMP2-treated and untreated groups (Figure 7, C and D, and Supplemental Figure 9). Importantly, both rhTIMP2 and its mutant rhAlaTIMP2 failed to reduce the phosphorylation of VE-cadherin in HBMECs with α_3_ integrin knockdown (Figure 7, E and F). Together, these data indicate a linear α_3_ integrin/p-Src/p-VEC pathway that regulates endothelial barrier integrity downstream of TIMP2.

### TIMP2 stabilizes VE-cadherin/catenin complex and VE-cadherin membrane localization.

**Figure 8:**
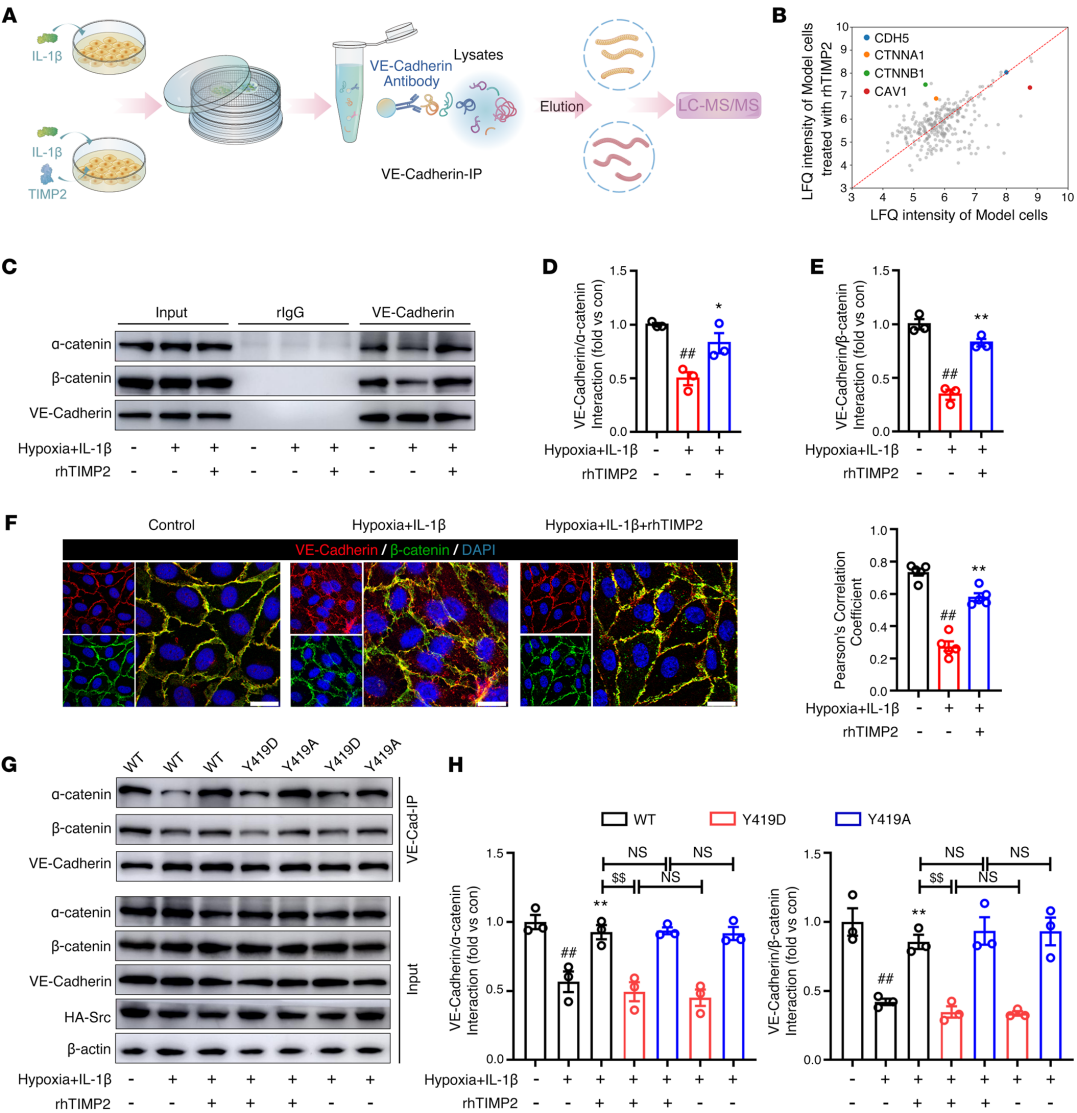
TIMP2 reduces hypoxic plus IL-1β injury–induced VE-cadherin/catenin complex destabilization through Src inhibition. (**A**) Schematic representation outlining the procedure for the identification of VE-cadherin–interacting molecules regulated by TIMP2. (**B**) Quantitative LC-MS analysis of VE-cadherin–binding proteins after HBMEC treatment with or without rhTIMP2 in HBMECs subjected to hypoxia plus IL-1β injury for 24 hours. LFQ,label-free quantitation. (**C**–**F**) HBMECs treated with rhTIMP2 or PBS were subjected to hypoxia plus IL-1β injury for 24 hours. Total cell lysates from the indicated groups were extracted and subjected to IP assay using VE-cadherin antibody. Western blot analysis (**C**) and quantification (**D** and **E**) were performed for the interaction between VE-cadherin and α-catenin, or VE-cadherin and β-catenin. Data represent 3 independent experiments. (**F**) Immunofluorescence staining and quantitative analysis of colocalization of VE-cadherin (red) and β-catenin (green) in the indicated groups. Cells were counterstained with Hoechst 33342 (blue) for nuclear labeling. Scale bars: 20 μm. Data represent 5 independent experiments. (**G** and **H**) HBMECs transfected with WT, Y419D, or Y419A Src were treated with rhTIMP2 or PBS, and then subjected to hypoxia plus IL-1β injury for 24 hours. Western blot analysis (**G**) and quantification (**H**) were performed for the interaction between VE-cadherin and α-catenin, or VE-cadherin and β-catenin. Data represent 3 independent experiments. ^##^*P* < 0.01 vs. control group, *P < 0.05 and **P < 0.01 vs. hypoxia plus IL-1β group, ^$$^*P* < 0.01, by 1-way ANOVA.

Phosphorylation of the cytosolic tail interrupts the interaction between VE-cadherin and its partner, which in turn disrupts cell-cell contacts. A large number of accessory molecules have been identified in the VE-cadherin interactome (17). To determine the mechanisms by which VE-cadherin regulates the overall architecture of the EC barrier during TIMP2 treatment, we performed quantitative LC-MS analysis following IP with an anti–VE-cadherin antibody to identify proteins that preferentially interacted with VE-cadherin in the rhTIMP2-treated group compared with the group exposed only to hypoxic plus inflammatory injury (Figure 8A). As shown in Figure 8B, VE-cadherin levels were similar between the 2 groups, indicating that the experimental data were reasonable. Among the identified proteins, α-catenin and β-catenin were expressed at substantially higher levels in the rhTIMP2-treated group than in the hypoxia plus IL-1β–induced model group. It has been reported that β-catenin interacts with the cytoplasmic region of VE-cadherin and binds α-catenin, forming the VE-cadherin/catenin complex, which is critical in the maintenance of endothelial monolayer integrity (51). In addition, the interaction between VE-cadherin and β-catenin is regulated by the phosphorylation of VE-cadherin on tyrosine 731, which is dependent on Src activation (48). Therefore, we next investigated whether TIMP2 stabilizes the VE-cadherin/catenin complex in TBI. Western blot analysis revealed that hypoxia plus IL-1β decreased the binding of VE-cadherin and β-catenin as well as α-catenin in the membrane fraction, whereas the interaction was restored by exogenous rhTIMP2 treatment (Figure 8, C–E). A similar trend was observed for rhAlaTIMP2 treatment (Supplemental Figure 10, A–C). Consistent with the Western blot results, immunostaining confirmed that rhTIMP2 and rhAlaTIMP2 could promote the association of VE-cadherin and β-catenin (Figure 8F and Supplemental Figure 10D). Performing co-IP experiments in HBMECs overexpressing Src WT and mutants, we observed that overexpression of the Y419D Src mutant, but not the Y419A mutant, significantly abolished the TIMP2-mediated promotion of the interaction between VE-cadherin and α-/β-catenin (Figure 8, G and H, and Supplemental Figure 10, E and F). Similar results were also observed using immunostaining assays (Supplemental Figure 10G). Collectively, these data suggest that in HBMECs exposed to hypoxic plus inflammatory insult, TIMP2 stabilizes the VE-cadherin/catenin complex, in which Src activation is specifically needed.

**Figure 9:**
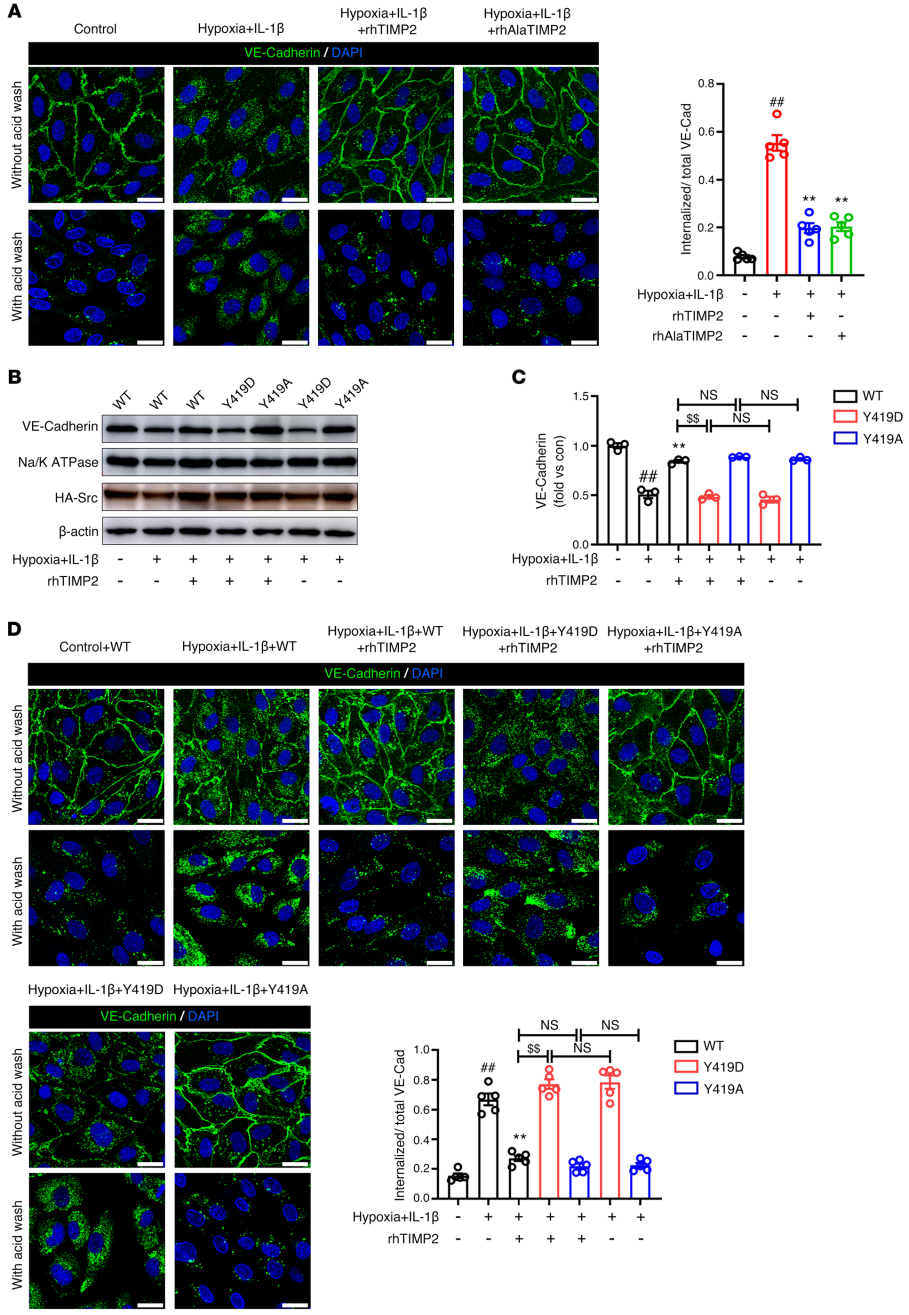
TIMP2 decreases hypoxic plus IL-1β injury–induced VE-cadherin internalization through Src inhibition. (**A**) HBMECs treated with rhTIMP2, rhAlaTIMP2, or PBS were subjected to hypoxia plus IL-1β insult for 24 hours. VE-cadherin internalization from the indicated groups was detected by antibody feeding assays combined with immunofluorescence staining analyses. Representative images and quantification of the mean fluorescence intensity (MFI) of VE-cadherin from the indicated groups (*n* > 500 cells per group). Scale bars: 20 μm. Data represent 5 independent experiments. (**B**–**D**) HBMECs transfected with WT, Y419D, or Y419A Src were treated with rhTIMP2 or PBS, and then subjected to hypoxia plus IL-1β injury for 24 hours. Western blot analysis (**B**) and quantification (**C**) of VE-cadherin in the membrane fraction. (**D**) Representative images and quantification of the MFI of VE-cadherin from the indicated groups (*n* > 500 cells per group). Scale bars: 20 μm. Data represent 3 (**B** and **C**) or 5 (**D**) independent experiments. ^##^*P* < 0.01 vs. control group, **P < 0.01 vs. hypoxia plus IL-1β group, ^$$^*P* < 0.01, by 1-way ANOVA.

The phosphorylated cytosolic tail and disrupted interaction with catenins induced mislocalization of VE-cadherin (52). Using antibody feeding assays, we observed a marked increase in the internalization of VE-cadherin in hypoxic and inflammatory–injured HBMECs, which could be efficiently inhibited by exogenous addition of rhTIMP2 and rhAlaTIMP2 (Figure 9A). Again, Western blot analysis revealed that in HBMECs expressing the Y419D mutant, TIMP2 failed to rescue VE-cadherin internalization (Figure 9, B and C, and Supplemental Figure 11, A and B). In line with this, immunostaining analysis showed that the inhibiting effects of TIMP2 on VE-cadherin translocation were completely reversed by Y419D mutant expression (Figure 9D and Supplemental Figure 11C). Importantly, using an antibody against HA tag in antibody feeding assays, we observed increased translocation of VEC-3mu from the membrane to the cytosol and the inability to reverse VEC-3mu internalization by TIMP2 treatment (Supplemental Figure 12), further confirming that VE-cadherin phosphorylation is critical for its internalization and plays an essential role in the TIMP2-mediated barrier integrity pathway.

Together, the above data suggest that TIMP2 suppresses the hypoxic plus inflammatory insult–induced increase in EC permeability by regulating VE-cadherin/catenin complex stabilization and VE-cadherin internalization, and that inhibition of Src activation is specifically required for these effects.

### TIMP2 regulates Rac1 activity to attenuate stress fiber formation.

**Figure 10:**
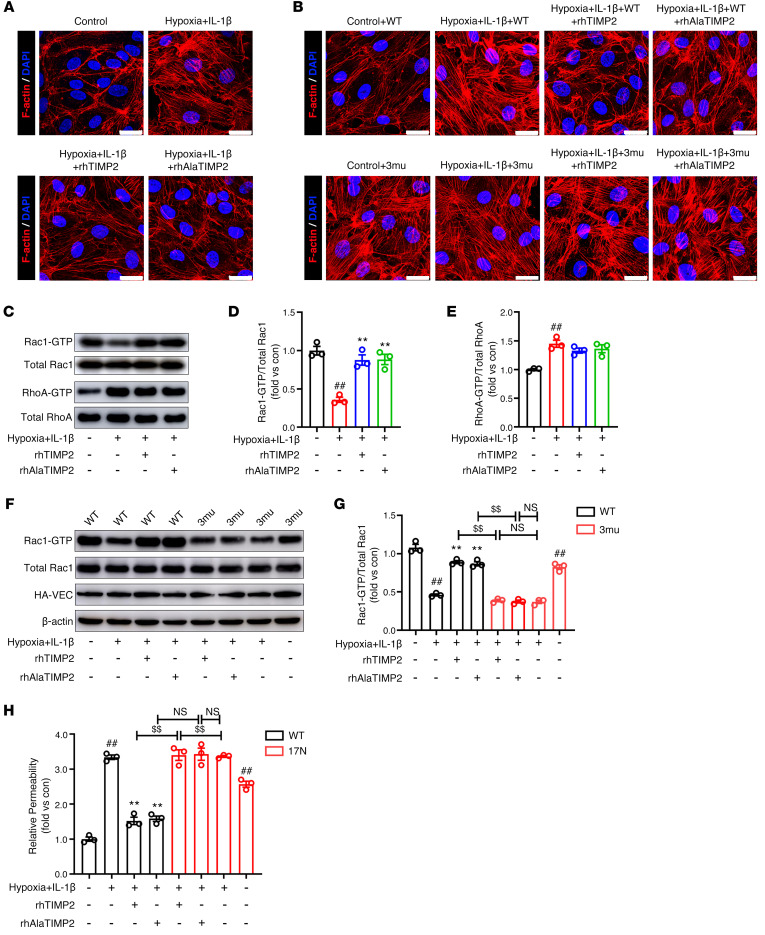
TIMP2 enhances Rac1 activity to attenuate stress fiber formation. (**A**) HBMECs treated with rhTIMP2, rhAlaTIMP2, or PBS were subjected to hypoxia plus IL-1β insult for 24 hours. Cells were double-labeled for F-actin^+^ (red) and the nuclear marker Hoechst 33342 (blue). (**B**) HBMECs transfected with WT or Y658&685&731E VE-cadherin were treated with rhTIMP2, rhAlaTIMP2, or PBS, and then subjected to hypoxia plus IL-1β insult for 24 hours. Cells were double-labeled for F-actin^+^ (red) and the nuclear marker Hoechst 33342 (blue). Scale bars: 25 μm. (**C**–**E**) HBMECs treated with rhTIMP2, rhAlaTIMP2, or PBS were subjected to hypoxia plus IL-1β insult for 24 hours. Western blot analysis (**C**) and quantification of Rac1-GTP levels (**D**) and RhoA-GTP levels (**E**) in cell lysates. (**F** and **G**) HBMECs transfected with WT or Y658&685&731E VE-cadherin were treated with rhTIMP2, rhAlaTIMP2, or PBS, and then subjected to hypoxia plus IL-1β injury for 24 hours. Western blot analysis (**F**) and quantification (**G**) of Rac1-GTP levels. (**H**) HBMECs transfected with WT or 17N Rac1 were treated with rhTIMP2, rhAlaTIMP2, or PBS, and then subjected to hypoxia plus IL-1β injury for 24 hours. Transwell permeability assays were performed to assess EC barrier integrity. Data represent 3 independent experiments. ^##^*P* < 0.01 vs. control group, **P < 0.01 vs. hypoxia plus IL-1β group, ^$$^*P* < 0.01, by 1-way ANOVA.

In ECs, the VE-cadherin/catenin complex is not only the backbone of adherens-type junctions but also activates signaling molecules with roles in actin filament dynamics to ensure junctional adhesion and barrier function (53). Moreover, the Gene Ontology Cellular Component analysis of TIMP2 IP-MS results showed that TIMP2 interacts with several cytoskeleton components (Figure 4A). Therefore, we next focused on whether actin filament rearrangement is involved in TIMP2-mediated EC barrier integrity. Using fluorescent phalloidins to label actin filaments, we observed that hypoxic plus inflammatory insult enhanced contractile actin bundles in cultured HBMECs, as manifested by increased formation of the cytoplasm traversing stress fibers. The addition of exogenous rhTIMP2 and rhAlaTIMP2 both reversed the stress fibers to junction-associated circumferential actin bundles under the cell surface, confirming the ability of TIMP2 to inhibit stress fiber formation in ECs after TBI (Figure 10A). Additionally, rhTIMP2 and rhAlaTIMP2 displayed no effect on stress fiber formation in HBMECs expressing either the VEC-3mu (Figure 10B) or the Y419D Src mutant (Supplemental Figure 13A), indicating that regulation of actin filament rearrangement occurs downstream of TIMP2-mediated VE-cadherin/catenin complex stabilization.

Actin cytoskeleton dynamics are centrally regulated by Rho GTPase family members, among which Rac1 and RhoA are the most strongly associated with junction-related actin reorganization in ECs (54). GST fusions of the p21-binding domain of PAK and the Rho-binding domain of rhotekin were used to measure levels of GTP-bound Rac1 (activated Rac1) and GTP-bound RhoA (activated RhoA), respectively, via pull-down assays. As shown in Figure 10, C and D, hypoxic plus inflammatory insult clearly reduced the level of Rac1-GTP, which was significantly upregulated by rhTIMP2 and rhAlaTIMP2 treatments. However, rhTIMP2 and its mutant had little inhibitory effect on the marked increase in RhoA activity in HBMECs subjected to the hypoxia plus IL-1β (Figure 10, C and E), indicating that TIMP2 mainly inhibits stress fiber formation by regulating Rac1 activity. It has been reported that stabilization of VE-cadherin on the membrane is essential for the recruitment of upstream effectors to activate Rac1 (55, 56). We examined Rac1 activity in HBMECs overexpressing WT VE-cadherin or VEC-3mu. As expected, both rhTIMP2 and its mutant failed to increase Rac1-GTP levels in HBMECs transfected with VEC-3mu, which was unable to localize to the membrane in HBMECs exposed to hypoxic plus inflammatory injury (Figure 10, F and G). Furthermore, we observed that silencing α_3_ integrin suppressed rhTIMP2-induced Rac1 activation (Supplemental Figure 13, B and C), and overexpression of T17N Rac1 (a dominant-negative mutant unable to bind GTP) but not WT Rac1 strongly abrogated the ability of rhTIMP2 and rhAlaTIMP2 to rescue HBMEC paracellular permeability (Figure 10H). These results collectively indicated that α_3_ integrin/p-Src/p-VEC/Rac1 is the downstream pathway of TIMP2-mediated EC barrier integrity.

## Discussion

**Figure 11:**
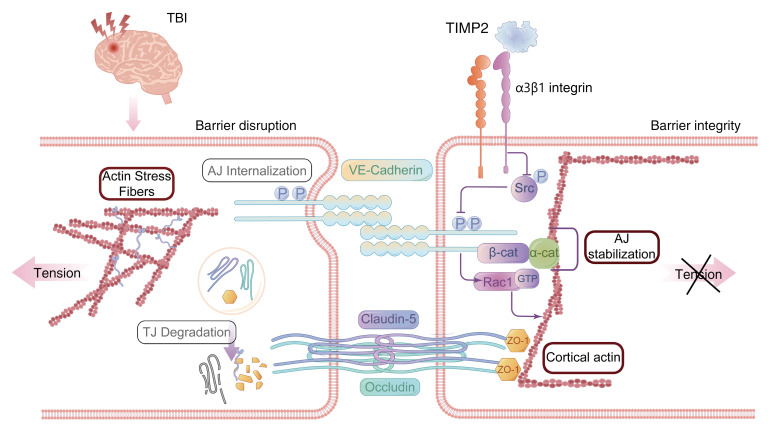
A working model depicting the role of TIMP2 in regulating BBB integrity in TBI.

The present study uncovered the ability of TIMP2 to protect against BBB disruption in an experimental TBI model and its underlying regulatory mechanism. Functionally, TIMP2 attenuates BBB leakage and EC barrier hyperpermeability in in vivo and in vitro TBI models, respectively. Mechanistically, TIMP2 interacts with α_3_β_1_ integrin on ECs to inhibit Src-dependent VE-cadherin phosphorylation and internalization. Additionally, stabilization of VE-cadherin on the membrane is indispensable for the effect of TIMP2 on EC barrier integrity, contributing to enhanced Rac1 activity to counterbalance actin filament reorganization and regulate overall junctional complex expression and distribution (Figure 11).

TIMP2 is a secreted protein that was originally identified as an endogenous inhibitor of MMPs. In addition to regulating proteolytic activity, TIMP2 directly interacts with cell surface receptors to exert pluripotent effects on cell proliferation, differentiation, and migration through MMP-independent mechanisms (25, 57, 58). In the CNS, TIMP2 expression is enriched in neuronal progenitors (59), promoting neuronal differentiation and neurite outgrowth (31). TIMP2 has also been implicated in neurological disorders. TIMP2 levels are elevated in a variety of CNS diseases, including multiple sclerosis, Alzheimer’s disease, and Huntington’s disease (60–62). Overexpression of TIMP2 exhibits antiinflammatory and neuroprotective effects, inhibits development of experimental autoimmune encephalomyelitis (34), attenuates ischemic brain injury (35), and alleviates cognitive deficits in aged mice (37). Although the neuroprotective effects of TIMP2 have been attributed to its MMP-inhibiting activity, synthetic MMP inhibitors are unable to mimic these beneficial effects. Considering the lack of FDA-approved clinical interventions and the urgent need for new therapeutics for TBI, we explored the protective effect of TIMP2 in mice with experimental TBI. We used mutagenesis to generate AlaTIMP2, a mutant form of TIMP2 without MMP-inhibiting activity (20), and demonstrated that TIMP2 contributes to protecting against neurological deficits and BBB breakdown in TBI in a nonproteolytic manner.

A number of previous studies have revealed that TIMP2 is associated with the biological functions of ECs, especially the inhibition of mitogenic growth factor–stimulated angiogenesis by binding to α_3_β_1_ integrin (22) or ILGF-1R (21). The interaction between TIMP2 and α_3_β_1_ integrin increases Shp-1 binding to RTKs, resulting in an altered phosphorylation pattern that affects downstream signaling pathways involved in various cellular responses. Using IP-MS analysis, we identified α_3_β_1_ integrin as the receptor for TIMP2 on ECs. More importantly, we also found that the α_3_ subunit is required for the formation of the TIMP2/α_3_β_1_ integrin complex. To further investigate the role of α_3_ integrin in the effect of TIMP2 on EC barrier integrity in vivo, we knocked down α_3_β_1_ integrin specifically in brain ECs with the AAV-BR1 system (40). As expected, TIMP2 failed to reverse TBI-induced BBB leakage and neurological abnormalities in AAV-shITGA3 mice as effectively as in AAV-shNC mice, confirming that the association of TIMP2 with α_3_β_1_ integrin on ECs is indispensable for its ability to protect the BBB.

High sealing efficiency via the junctional complex is a unique feature of the EC barrier. The degradation and aberrant distribution of JPs markedly increase paracellular permeability, thus allowing the passage of blood components into the brain parenchyma. The present findings show that TIMP2, upon binding to α_3_β_1_ integrin, inhibits the impaired paracellular pathway of the BBB by preventing TJ degradation and AJ translocation. The AJ component VE-cadherin is an EC-specific member of the cadherin family that plays an essential role in controlling vascular permeability (46). Obvious collapse of the vascular system in VE-cadherin–knockout mice leads to embryonic lethality (63). Blocking antibodies against VE-cadherin significantly increase pulmonary and cardiac vascular permeability and lead to the accumulation of inflammatory cells (64). VE-cadherin also regulates the expression and localization of TJ proteins, including claudin-5 and occludin, and is therefore responsible for the entire EC barrier architecture (65, 66). Notably, our data show that the localization of VE-cadherin at the plasma membrane is essential for TIMP2-mediated maintenance of BBB integrity in TBI.

The nonreceptor tyrosine kinase Src has been demonstrated to be the critical downstream effector of integrin complexes. Inflammatory mediators induce Src phosphorylation at Tyr419, a defining signature of Src activation (67). Both in vivo and in vitro, TIMP2 inhibits Src phosphorylation in ECs induced by TBI. The necessity of Src inhibition was also demonstrated by the fact that EC permeability was increased after treatment of cells with the constitutively active Src variant Y419D. Src activation induces VE-cadherin tyrosine phosphorylation, leading to weakened junctions and increased vascular permeability. Multiple tyrosine sites in the cytoplasmic tail of VE-cadherin, including Y658, Y685, and Y731, have been reported to be regulated by Src activation (15, 48, 49). In this study, we introduced several combinations of VE-cadherin phosphorylation mimic variants and observed that only the triple mutations (Y658&685&731E) could completely abolish the beneficial effects of TIMP2 on EC barrier integrity. Additionally, our data also showed that TIMP2-mediated Src inhibition is required to reduce VE-cadherin phosphorylation and internalization.

VE-cadherin–based cell-cell junctions are strengthened by anchoring to the junction-associated actin bundle. A large number of proteins have been identified as scaffolds that bridge the interaction between VE-cadherin and actin filaments (53). Using quantitative IP-MS, we identified α-catenin and β-catenin as intracellular linker molecules that preferentially interacted with VE-cadherin in the TIMP2-treated group compared with the model group. VE-cadherin binds to β-catenin through its cytoplasmic tail, and in turn binds to α-catenin, which is fundamental to engage the cytoskeleton. It has been reported that phosphorylation of VE-cadherin at tyrosine residues in the cytoplasmic domain results in reduced binding to partners, thereby triggering actin cytoskeleton rearrangement and disrupting EC junctions (16, 52). In our study, overexpression of the active Src mutant Y419D, which is known to enhance VE-cadherin phosphorylation, abolished the stabilizing effect of TIMP2 on the VE-cadherin and β-catenin complex in TBI and disrupted the actin network. Under physiological conditions, a cortical rim, which serves as an important anchor for cell-cell adhesions, forms in ECs (68). When homeostasis is disrupted in response to permeability-inducing stimuli, there is a formation of radial stress fibers that break intercellular junction complexes, including loosening VE-cadherin/catenin binding to destabilize VE-cadherin on the membrane and subsequently trigger internalization. In the present study, we demonstrated that TIMP2 inhibits VE-cadherin internalization. The homophilic interactions between VE-cadherin molecules from adjacent ECs recruit accessory molecules to the intracellular tail to activate Rac1 (56), a barrier-stabilizing mediator that promotes the assembly of cortical actin bundles adjacent to the junction and thereby stabilizes EC cell-cell contacts (69). Consistent with this, TIMP2 treatment markedly increased Rac1-GTP levels, thereby enhancing circumferential actin bundles and attenuating perpendicular contraction bundle formation. Importantly, the necessity of Rac1 activation was further confirmed by the fact that TIMP2 did not inhibit hyperpermeability in HBMECs expressing mutant Rac1-17N.

Taken together, our findings reveal that TIMP2 preserves BBB integrity in TBI through an MMP-independent mechanism. TIMP2 interacts with α_3_β_1_ integrin on ECs to prevent VE-cadherin phosphorylation through inhibiting Src activation, thereby stabilizing the VE-cadherin/catenin complex and VE-cadherin membrane localization. Moreover, a reduction in VE-cadherin internalization contributes to Rac1 activation, preventing stress fiber formation. The protective role of TIMP2 in TBI implies that TIMP2 has the potential to be a therapeutic agent for CNS disorders involving BBB sealing deficiency and that the α_3_β_1_ integrin/Src/VEC/Rac1 pathway regulated by TIMP2 is a potential therapeutic target for alleviating BBB damage.

## Methods

Additional methods are provided in Supplemental Methods.

### Animals

Male C57BL/6N mice were obtained from Vital River Laboratories Technology Co. Ltd. at 8 weeks of age. Animals were housed 3 per cage in a temperature-controlled (21°C–25°C) and humidity-controlled (45%–50%) room with a 12-hour light/12-hour dark cycle and were fed a standard rodent diet with water ad libitum.

### Expression and purification of recombinant TIMP2 proteins

The pcDNA3.1B-rhTIMP2/rhAlaTIMP2/rmTIMP2/rmAlaTIMP2-myc-His plasmid was constructed and transfected into HEK293T cells (American Type Culture Collection) using polyethyleneimine (40816ES02, Yeason Biotechnology). The medium was changed to DMEM (10564011, Thermo Fisher Scientific) without serum at 16 hours after transfection. Culture supernatants containing secreted TIMP2 protein were collected on the sixth day after transfection and concentrated by ultrafiltration. Recombinant murine TIMP2 (rmTIMP2) or its mutant rmAlaTIMP2 and recombinant human TIMP2 (rhTIMP2) or its mutant rhAlaTIMP2 were purified using HisPur Ni-NTA resin (88221, Thermo Fisher Scientific) with a histidine tag, eluted with 500 mM imidazole, and dialyzed into PBS (pH 7.4) by centrifugation using a 3 kDa molecular mass cutoff filter (UFC900396, Millipore). The purified recombinant TIMP2 proteins were aliquoted and stored at –80°C.

### Surgical procedures and rTIMP2 administration

Mice anesthetized with isoflurane were cannulated and placed in a stereotaxic frame. A craniectomy with a 3 mm diameter was performed 0.8 mm posterior to the bregma and 1.3 mm lateral to the sagittal suture in the right hemisphere, with a portable drill to remove the bone flap. Using a modified Feeney weight drop injury (WDI) model, TBI was induced by advancing a 2-mm-impacting rod into the exposed right parietal cortex, and then dropping a 20 g weight from a height of 25 cm. Sham-injured mice were not subjected to impact but otherwise underwent similar surgical procedures. After surgery, mice were promptly intravenously given rmTIMP2, rmAlaTIMP2, or PBS and housed under the conditions described above.

### Neurological function assessments

A battery of functional tests, including the rotarod motor test, beam walk test, and modified neurological severity score (mNSS), were performed by observers unaware of the experimental treatments at 24, 48, and 72 hours after the TBI surgery. Mice were pretrained for 3 days for the rotarod and beam walk tests.

#### Rotarod test.

An accelerating rotarod test was performed to estimate the motor function of the TBI mice. The rotation speed was gradually increased at a constant rate from 4 rpm to 35 rpm over 3 minutes. Mice were placed on a 3.5 cm rotating rod to record fall latency for 3 trials.

#### Beam walk test.

The beam walk test was used to assess motor coordination, particularly of the hind limb. Mice were placed on a 6-mm-wide, 1-m-long narrow wooden beam that was 30 cm above the ground. The hind-limb performance traversing the beam (based on a 1–7 rating scale) was recorded as previously described (70).

#### Modified neurological severity score.

The mNSS, a multifunctional evaluation scale consisting of motor, reflex, sensory, and balance tests, was used to assess the neurological injury of mice after TBI. Neurological function is graded on a scale of 0 to 18. Higher scores represent more severe neurological deficits (70).

### Evans blue extravasation

A 4% Evans blue solution (E2129, Sigma-Aldrich) was injected into the tail vein at 3 mL/kg and allowed to circulate for 2 hours. Mice anesthetized with isoflurane were intracardially perfused with sterile saline to remove dye from the circulation. Evans blue dye was extracted by incubation of brain tissues in 1 mL formamide at 45°C for 48 hours, and the concentration was determined at 620 nm with a Universal Microplate Reader (BioTek Instruments).

### Virus injection

To knock down α_3_ integrin in brain vascular ECs in vivo, 4-week-old mice were intravenously injected with adeno-associated virus (AAV)–Br1-FLAG-shNC or AAV-Br1-FLAG-shITGA3 (Hanbio Biotechnology) at a dose of 2.0 **×** 10^11^ genomic particles per mouse. The sequence of α_3_ integrin shRNA in Br1–α_3_ integrin–RNAi is 5′-GGTGCCGTCTATGTCTTCA-3′. Further detections were performed 3 weeks after virus injection.

### Cell culture and reagents

HBMECs from Cell Systems (ACBRI376) were cultured in complete endothelial cell medium (ECM; 1001, ScienCell) at 37°C in a humidified atmosphere of 5% CO_2_. Confluent HBMECs were treated with 20 ng/mL IL-1β plus hypoxia. The hypoxic environment was established by placement of the HBMECs in a hypoxia incubator chamber (27310, Stemcell) and purging of the chamber with a 95% N_2_ and 5% CO_2_ gas mixture at 20 mL/min for 10 minutes. The chamber was sealed and kept at 37°C for 24 hours.

### Transfection with cDNA or small interfering RNA

HBMECs were transfected with Src constructs (WT, Y419D, Y419A), VE-cadherin constructs (WT, Y685E, Y658&731E, Y658&685&731E), or Rac1 constructs (WT, T17N) using jetOPTIMUS (101000006, Polyplus) following the manufacturer’s instructions. The siRNAs against β_1_ integrin (siβ1, 5′-CCGUAGCAAAGGAACAGCA-3′), α_3_ integrin (siα3, 5′-GUGUACAUCUAUCACAGUA-3′), and the nonsilencing control RNA (NC, 5′-UUCUCCGAACGUGUCACGU-3′) were purchased from GenePharma Corp. The siRNAs and NC were transfected into cell lines using jetPRIME (101000046, Polyplus) following the manufacturer’s instructions. The medium was changed 6 hours after transfection. HBMECs were cultured for an additional 48–60 hours before further assays.

### In vitro transendothelial permeability assay

The in vitro BBB model composed of a monolayer of HBMECs or the 3D BBB model composed of primary cells was exposed to hypoxia plus 20 ng/mL IL-1β for 24 hours. To assess paracellular permeability, 1 mg/mL 70 kDa FITC-dextran (FD70S, Sigma-Aldrich) was added to the luminal chamber. After 2 hours, 40 μL medium was collected from the abluminal chamber, and the fluorescence intensity was measured using a fluorescence reader (485 nm excitation and 528 nm emission).

### VE-cadherin internalization assay

VE-cadherin internalization was evaluated using an antibody feeding assay as previously described (15). Briefly, HBMECs from the indicated groups were incubated with an antibody against the VE-cadherin extracellular domain (sc-52751, Santa Cruz Biotechnology; 10 μg/mL) or HA tag (sc-57592, Santa Cruz Biotechnology; 5 μg/mL) at 4°C for 1 hour in ECM basal medium containing 3% BSA without supplements. Unbound antibody was removed by rinsing of cells with ice-cold ECM. Cells were treated with rhTIMP2, rhAlaTIMP2, or PBS and then subjected to hypoxia plus IL-1β injury. Cells were acid-washed (ECM containing 2 mM glycine and 3% BSA at pH 2.7) for 8 minutes to remove antibody from the cell surface, followed by fixation with 2% paraformaldehyde for 10 minutes at room temperature, and processed for immunofluorescence.

### Western blot analysis

Whole-cell lysates were prepared using RIPA lysis buffer (50 mM Tris-HCl, pH 7.5, 150 mM NaCl, 1% Triton X-100, 1% sodium deoxycholate, 0.1% SDS, 10 mM EDTA) supplemented with complete EDTA-free protease inhibitor cocktail (4693116001, Roche) and phosphatase inhibitors (04906845001, Roche). Protein samples were separated by SDS-PAGE and then transferred electrophoretically onto PVDF membranes (ISEQ00010, Millipore). The membranes were incubated overnight at 4°C with primary antibodies. Anti-mouse or anti-rabbit IgG-HRP was used as a secondary antibody for 1 hour at room temperature, and signals were detected using ImageQuant LAS 4000 (GE Healthcare). The antibodies used are listed in Supplemental Table 2.

### Immunofluorescence staining

Cells fixed with 4% paraformaldehyde were blocked with 10% normal goat serum containing 0.1% Triton X-100 for 1 hour at room temperature and stained with the primary antibodies overnight at 4°C. On the second day, the cells were incubated with the corresponding Alexa Fluor–conjugated secondary antibodies for 1 hour and counterstained with Hoechst 33342 (H1399, Invitrogen) for 15 minutes at room temperature. F-actin staining was performed using rhodamine-conjugated phalloidin for 2 hours at room temperature. Images were captured and analyzed using a Leica TCS SP8 confocal microscope (Leica Microsystems).

For immunofluorescence staining of paraffinized sections, the brain tissue sections were deparaffinized in Histo-Clear (64111-01, Electron Microscopy Sciences) and rehydrated through a graded ethanol series. The sections were pretreated using heat-mediated antigen retrieval with EDTA-Tris buffer (pH 9.0) and blocked with 10% goat serum at room temperature for 1 hour, followed by incubation with primary antibodies overnight at 4°C. The procedures that followed were identical to those described above. The antibodies used are listed in Supplemental Table 2.

### Cell surface biotinylation and isolation

HBMECs were incubated with ice-cold 0.5 mg/mL cell membrane nonpermeable EZ-Link Sulfo-NHS-LC-Biotin (sulfosuccinimidyl-6-[biotin-amido] hexanoate) (21335, Pierce) in Ca^2+^/Mg^2+^-PBS (0.01 M PBS containing 1 mM CaCl_2_ and 0.5 mM MgCl_2_, pH 7.5) for 30 minutes at 4°C for surface biotinylation, followed by incubation with 100 mM glycine in TBS for 10 minutes at 4°C to quench the residual biotin. Cells were lysed in ice-cold NP-40 lysis buffer (50 mM Tris-HCl, pH 7.5, 150 mM NaCl, 1% NP-40) with protease inhibitor cocktail, and the cleared extracts were incubated with 20 μL of neutravidin beads (29202, Pierce) on a rotator for 6 hours at 4°C. The cell surface proteins captured by the beads were eluted with SDS buffer and assayed by Western blot analysis.

### Immunoprecipitation

HBMECs were lysed in RIPA lysis buffer or NP-40 lysis buffer containing protease and phosphatase inhibitors on ice for 30 minutes. Cleared extracts (800 μg per sample) were incubated with protein G magnetic beads (10003D, Invitrogen) conjugated with primary antibody or isotype control antibody at 4°C overnight with rotation. The beads were washed 4 times with corresponding lysis buffer to remove nonspecific proteins, and then the bound proteins were eluted in SDS buffer for Western blot analysis.

### Rac1/RhoA activation assay

Rac1/RhoA activity was assessed using the RhoA/Rac1/Cdc42 Activation Assay Combo Biochem Kit (BK030, Cytoskeleton) according to the manufacturer’s instructions. Briefly, HBMECs were lysed in the provided lysis buffer supplemented with protease inhibitor cocktail. The cleared extracts were immediately incubated with PAK-PBD– or rhotekin-RBD–conjugated agarose beads, which preferentially pulled down GTP-bound active Rac1 or RhoA. After incubation at 4°C on a rotator for 1 hour, the beads were washed with wash buffer and centrifuged at 3,000*g* for 2 minutes. The active Rac1/RhoA proteins were eluted in 2× Laemmli sample buffer and detected by Western blot analysis using a Rac1/RhoA-specific antibody.

### Statistics

All grouped data are presented as the mean ± SEM. The number of animals and experimental replicates is indicated in the figure legends. Differences between groups were assessed by 1-way ANOVA with Tukey’s multiple-comparison test. GraphPad Prism 7.00 software (RRID: SCR_000306) was used for all statistical analyses. A *P* value less than 0.05 was considered significant.

### Study approval

All animal care and experimental procedures were in accordance with the Animal Management Rule of the Ministry of Health of the People’s Republic of China (Document 55 of 2001) and approved by the Laboratory Animal Ethics Committee of the Chinese Academy of Medical Sciences.

### Data availability

Values for all data points in graphs are reported in the Supporting Data Values file.

## Author contributions

JT and YP designed the research studies. JT, YK, and SW performed the in vivo experiments. JT, YZ, and NS conducted the majority of the biological experiments. JT, XL, HW, and JL conducted the data analyses. YP conceived and supervised the project. JT drafted the manuscript. LW and YP revised the manuscript. All the authors read and approved the final manuscript.

## Supplementary Material

Supplemental data

Supplemental data set 1

## Version 1 11/28/2023

In-Press Preview

## Version 2 02/01/2024

Electronic publication
